# Patterns of Self-Reported Occupational Stress Experienced by Lithuanian Police Officers: A Cross-Sectional Study

**DOI:** 10.3390/healthcare13233077

**Published:** 2025-11-26

**Authors:** Birute Strukcinskiene, Jonas Jurgaitis, Rasa Grigoliene, Dovile Karoblyte, Erika Zuperkiene, Dalia Martisauskiene, Zydrune Gedvile, Gintautas Virketis, Linas Venclauskas, Agnieszka Genowska

**Affiliations:** 1 Department of Public Health, Faculty of Health Sciences, Klaipeda University, LT-92294 Klaipeda, Lithuania; jonas.jurgaitis@ku.lt (J.J.); dovile.karoblyte@ku.lt (D.K.); dalia.martisauskiene@ku.lt (D.M.); zydrune.gedvile@edu.ku.lt (Z.G.); gintautas.virketis@ku.lt (G.V.); linas.venclauskas@ku.lt (L.V.); 2 Department of Informatics and Statistics, Faculty of Marine Technologies and Natural Sciences, Klaipeda University, LT-92294 Klaipeda, Lithuania; rasa.grigoliene@ku.lt; 3 Department of Management and Economics, Faculty of Social Sciences and Humanities, Klaipeda University, LT-92294 Klaipeda, Lithuania; erika.zuperkiene@ku.lt; 4 Department of Public Health, Medical University of Bialystok, 15-295 Bialystok, Poland

**Keywords:** occupational stress, police officers, work environment, stress at work, occupational health, mental health

## Abstract

Background/Objectives: Occupational stress among police officers has been widely studied internationally, yet little is known about how stressors manifest in smaller, post-transition European contexts such as Lithuania. The study aimed to identify key occupational stressors among Lithuanian police officers and to examine how stress patterns differ by gender, job position, and years of service, using a multidimensional framework of organizational and interpersonal factors. Methods: A cross-sectional survey was conducted in 2024–2025 among 381 police officers from five randomly selected Lithuanian police stations. Participants completed the validated Lithuanian version of the Health and Safety Executive Management Standards Indicator Tool (HSE-MSIT). Seven domains of occupational stress were analyzed: job demands, managerial and peer support, relationships, organizational change, work control, and role clarity. Statistical analyses included Mann–Whitney U test, univariate ANOVA, and confirmatory factor analysis (CFA) with multi-group invariance testing. Results: The CFA supported a second-order structure of job stress, although model fit indices showed moderate adequacy (CFI = 0.768, TLI = 0.751). Managerial support, change at work, and peer support were the strongest contributors to the overall stress construct. While gender-related differences were minimal, officers with longer tenure reported lower job demands and greater role clarity. Junior officers expressed a more positive perception of feedback from managers and organizational changes. Measurement invariance tests revealed some item-level non-invariance, suggesting that group comparisons should be interpreted with caution. Conclusions: Occupational stress among Lithuanian police officers is primarily shaped by organizational rather than individual factors. Enhancing managerial competence, communication transparency, and peer-support mechanisms could substantially improve officers’ psychological well-being and resilience within law enforcement institutions.

## 1. Introduction

Occupational stress refers to the strain that arises when professional duties outweigh an individual’s ability to cope, producing psychological, emotional, and physical consequences across many professional fields [1,2,3]. Researchers typically describe four broad categories of workplace demands. The first relates to tasks themselves—heavy workloads, insecure employment, or duties that are too complex often heighten stress. A second source is linked to professional roles: conflicting expectations or poorly defined responsibilities may leave employees uncertain and tense. Physical working conditions, such as temperature, lighting, or the adequacy of facilities, represent a third dimension, with unfavorable environments undermining comfort and performance. Finally, the social climate at work also matters; tensions with colleagues or supervisors, unsuitable leadership styles, or group pressure can all magnify stress when interpersonal relations are strained [4,5]. This classification of workplace stressors aligns with established theories of occupational stress.

According to the Job Demands–Resources (JD–R) model, stress arises when job demands—such as exposure to traumatic events, shift work, or high workload—outweigh available resources, including supportive leadership, peer support, or adequate recovery time [6]. In policing, this imbalance is frequently observed, as officers face unpredictable operational environments with limited organizational support. Similarly, the Effort–Reward Imbalance (ERI) model emphasizes that stress emerges when substantial professional effort, such as maintaining public safety under dangerous conditions, is not matched with adequate rewards like fair pay, recognition, or career progression [7,8]. Empirical studies confirm that both models are useful in explaining why police officers are particularly vulnerable to chronic stress, burnout, and related health risks [9].

In addition to these general workplace stressors, policing involves unique challenges that directly affect both physical and psychological well-being [6,10]. Officers are regularly exposed to traumatic events, hazardous situations, and rapidly changing environments. Irregular or rotating shifts disrupt circadian rhythms and lead to chronic fatigue, while public scepticism and negative attitudes toward the police add to emotional strain. Work–family conflict and limited social support also intensify stress levels. Other stressors include carrying heavy protective gear, making rapid decisions in life-threatening contexts, and managing distressing cases such as child abuse or fatalities. Lifestyle factors such as insufficient sleep and maladaptive coping strategies, including increased alcohol or tobacco use, are also commonly reported [11,12,13,14,15,16,17].

The COVID-19 pandemic magnified these long-standing stressors. Officers faced inconsistent leadership, constantly changing policy directives, and operational uncertainty, alongside higher infection risk and heavier workloads [18,19,20]. These conditions contributed to burnout, lower job satisfaction, thoughts of leaving the profession, and overall declines in well-being [21,22]. The European Commission’s Directorate-General for Health and Food Safety designated police officers, alongside healthcare workers, as part of the “critical workforce” during the pandemic, underscoring their heightened vulnerability to infectious disease, psychological strain, and physical exhaustion [19]. A multinational study, conducted in the Netherlands, Austria, Germany, Switzerland, and Spain, reported that nearly a quarter (23.2%) of officers experienced high infection risk, while job insecurity and frequent policy changes further undermined their work [23]. Together, these factors accelerated burnout and reduced quality of life [21,22].

Cross-national evidence consistently shows that police officers are at elevated risk of mental health problems, most notably depression and post-traumatic stress disorder (PTSD) [24]. For example, in Canada, 50.2% of federal officers and 36.7% of municipal officers met criteria for at least one psychological disorder. In Ethiopia, 28.9% of officers reported a mental health condition. In South Korea, over 40% were identified as being at high risk of PTSD, while in Malaysia 14.9% reported moderate occupational stress [11]. A large meta-analysis of studies conducted between 1980 and 2019, spanning 24 countries and 272,463 officers, reported prevalence rates of 14.6% for depression, 14.2% for PTSD, 9.6% for anxiety disorders, and 8.5% for suicidal ideation. Additionally, 5.0% of officers met criteria for alcohol dependence, while 25.7% engaged in hazardous drinking. Occupational stress was identified as the main contributing factor [14].

The effects of stress extend into family and private life. Work-family conflicts are frequent, particularly in households with dual earners [17,25]. An international study found that heightened vigilance outside work often translated into emotional strain within family dynamics. Women were more likely to assume childcare and domestic responsibilities, sometimes experiencing guilt about this imbalance, while men reported guilt for not contributing more at home. Shift work emerged as a paradoxical factor: on one hand, it could increase family contact, yet on the other, it complicated scheduling and raised stress [16].

In the United Kingdom, authors analyzed occupational stress and sickness absence. Within a year, over half of officers (54%) had missed work due to illness: 33% were absent for 1–5 days, 13% for 6–19 days, and 8% for 20 days or longer. Female officers who were divorced, single, smokers, or had mental health conditions were especially likely to report absence. Job dissatisfaction and high stress were strong predictors of absenteeism [26].

Physical health consequences are also significant. A study in Germany showed that 87.2% of officers reported high stress from organizational sources, and 88.4% considered their job overall stressful. Musculoskeletal pain was widespread: 50.4% reported back pain, 41.9% neck pain, and 33.8% shoulder pain. Carrying protective gear weighing up to 20 kg also aggravated these risks. Stress levels were further shaped by psychosocial factors such as low job satisfaction and high emotional demands [27]. Researchers reported similar findings in Spain, where 45.6% of officers experienced role uncertainty, 36.9% job dissatisfaction, and 35% recurrent health complaints such as headaches or musculoskeletal pain. Stress also encouraged unhealthy coping: 17.8% reported increased alcohol use and 14.6% increased tobacco use [28].

It was found that 54% of officers reported poor sleep quality, often linked to traumatic exposure and rotating shifts. Poor sleep was associated with less healthy diets: officers consumed fewer fruits and vegetables, with women reporting 32% lower intake compared to well-rested counterparts [23,29]. Authors observed that 92% of officers encountered firearms during duty, 86% had witnessed child abuse or neglect, and 73% had dealt with cases of child fatalities. Such exposures were closely tied to PTSD symptoms, with 40% of officers reporting intrusive memories, nightmares, and hypervigilance [12].

Policing is a profession marked by exceptionally high demands that affect not only officers’ physical health and psychological well-being but also their family and social lives. Elevated rates of depression, PTSD, anxiety, and sleep problems are well documented, while maladaptive coping and poor rest heighten cardiovascular and metabolic risks. Stress frequently spills over into private life, with shift work in particular shaping family dynamics and time allocation. Among the various sources of strain, organizational stressors—such as excessive workload, insufficient support, and limited decision-making autonomy—emerge as especially detrimental, undermining both health and operational performance.

While much is known from international research, cultural traditions, organizational culture, and national systems strongly influence how occupational stress is experienced and expressed. Over the past decade, the Lithuanian police system has undergone major reforms, including structural centralization, performance-based management, and expanded public accountability. These transformations, while improving efficiency and transparency, may have also intensified certain stressors by increasing administrative pressure, public scrutiny, and role ambiguity. Additionally, formal psychological support mechanisms for officers remain limited compared to many Western European systems.

In Lithuania, systematic research on police occupational stress remains scarce. Existing data largely focus on general well-being or burnout among civil servants rather than police-specific stressors. Little is known about how stress manifests across gender, job position, and years of service in this professional group. Therefore, this study aimed to explore the patterns of self-reported occupational stressors experienced by police officers in Lithuania.

The main objectives of the research were to identify the key occupational stressors experienced by police officers in Lithuania and to explore how these stressors differ by gender, length of service, and job role. By doing so, it seeks to provide evidence-based insights to guide tailored mental health initiatives, inform improvements in organizational practice, and support the development of more sustainable policing careers in the Lithuanian context. To address these research aims, a multidimensional validated instrument was applied to evaluate occupational stress domains, supported by advanced statistical modeling to test group differences and structural validity.

## 2. Materials and Methods

### 2.1. Study Design and Participants

The research was carried out in Lithuania between 2024 and 2025 as part of a cross-sectional survey among police officers working in five municipal police stations. The Republic of Lithuania is administratively divided into counties and municipalities. Currently, the country consists of ten counties and sixty municipalities. Counties are formed from territories of municipalities that share common social, economic, and ethno-cultural interests. To ensure territorial and institutional diversity, five police stations were selected using a purposive sampling approach, considering factors such as geographical coverage, commissariat size, and sociodemographic characteristics of employees. In each chosen commissariat, information about the ongoing research was publicly disseminated to ensure transparency and encourage participation.

Within each selected commissariat, stratified random sampling was applied to recruit participants proportionally by gender, length of service, and rank. During the study, approximately 7,300 police officers were registered in Lithuania, constituting the total population. Using Paniotto’s formula [30], with a 5% margin of error, the minimum required sample size was calculated as 364 respondents. In total, 381 officers completed the questionnaire, slightly exceeding the target. The sample included both men and women, as well as junior and senior officers, ensuring proportional representation.

The sampling process was randomized at the institutional level. All officers within the selected departments were invited to participate voluntarily, and all had an equal opportunity to be included in the study. All participants were provided with clear information about the purpose, scope, and procedures of the study. Data collection was conducted on-site using self-administered paper questionnaires.

Participation was voluntary, informed consent was obtained, and anonymity was guaranteed. Respondents were informed that only aggregated results would be reported. Completion of the questionnaire was considered as implied consent to participate. Permission to conduct the study was obtained from the heads of the participating institutions, and the relevant organizational ethics committee approved the research.

### 2.2. Measurement Tool

Occupational stress was assessed using the Health and Safety Executive Management Standards Indicator Tool (HSE-MSIT), originally developed by the United Kingdom’s Health and Safety Executive in 2004 to evaluate psychosocial risk factors at work. This instrument is internationally recognized and has been validated in multiple occupational settings as a reliable measure of work-related stressors. The Lithuanian version of the questionnaire was previously adapted and validated by the Lithuanian Institute of Hygiene, confirming its suitability for use in the national context.

The structure of the HSE-MSIT comprises 35 items organized into seven thematic domains: job demands (8 items), managerial support (5), peer support (4), relationships (4), change at work (3), work control (6), and role clarity (5). Items within the “demands” and “relationships” domains are reverse-scored, ensuring that higher scores consistently reflect more favorable psychosocial conditions and a lower risk of occupational stress.

Validation studies of the Lithuanian version confirmed satisfactory psychometric properties, with Cronbach’s alpha coefficients exceeding 0.70 across all domains and 0.82 for the overall scale, supporting the instrument’s reliability for use among Lithuanian police officers [31,32,33,34].

The instrument’s dimensional structure was subsequently examined using Confirmatory Factor Analysis (CFA) to verify the theoretical model and assess measurement invariance across gender, job position, and length of service. In addition, analysis of variance (ANOVA) was conducted to identify mean-level differences in stress domains between these groups.

### 2.3. Study Variables

The study collected socio-demographic information from participants, including their age and gender. Professional characteristics were also considered, with length of service divided into two groups (0–20 years and 21 years or more) and job position classified as either junior or senior.

In the Lithuanian police system, junior positions correspond to lower ranks such as patrol officers, junior investigators, community officers, or junior specialists, typically involving operational duties under supervision. Senior positions include higher ranks, such as senior investigators, inspectors, commissioners, and heads of units or divisions, as well as specialists with supervisory or coordinating responsibilities. The distinction reflects the formal career progression structure defined by the Lithuanian Police Department, where 20 years of service generally marks the transition to advanced professional or managerial roles.

Overall, the sample comprised 381 respondents, of whom 175 (45.9%) were men and 206 (54.1%) were women, 228 (59.8%) of respondents held junior positions and 153 (40.2%) senior positions, 205 (53.8%) with 0–20 years of service and 176 (46.2%) with 21 and over years of service, ensuring proportional representation of categories across different police units. The mean age of participants was 39.8 years (SD = 8.2), with most officers falling within the 31–45-year range. These socio-demographic and professional variables were used as grouping factors in subsequent analyses to explore potential differences in occupational stress patterns.

### 2.4. Statistical Analysis

Continuous variables were summarized with number of items, mean, median, standard deviation (SD), first and third quartile (Q1 and Q3, respectively) along with the range (min-max). Due to the lack of Global CFA model fit was evaluated using the chi-square test, the Comparative Fit Index (CFI) and Tucker–Lewis Index (TLI)—incremental indices that compare the target model to a null model, with higher values indicating better fit—together with the Root Mean Square Error of Approximation (RMSEA), which reflects population misfit per degree of freedom, and the Standardized Root Mean Square Residual (SRMR), which summarizes the average standardized residuals between observed and model-implied covariances. Conventional guidelines were applied for interpretation (good fit for CFI/TLI ≈ 0.95; RMSEA ≤ 0.08 and SRMR ≤ 0.08).

Due to the lack of normality in the distribution of variables (as verified by the Shapiro–Wilk test), non-parametric methods were applied. The Mann–Whitney U test was used to assess differences between two independent groups when the data did not meet the assumptions of parametric tests. This test is suitable for identifying distributional differences when variables are not normally distributed and observations are independent. Effect sizes were calculated to complement *p*-values and provide an estimate of the magnitude of observed effects or associations.

For each dependent variable (Demands, Managerial support, Peer support, Relationships, Change at work, Work control, Role clarity), a univariate ANOVA was fitted with three factors (Gender × Job position × Years of service, including interactions). Results are reported in corresponding tables as F statistics and *p*-values.

Then, a second-order confirmatory factor analysis (CFA) was specified, in which seven first-order latent factors (corresponding to the predefined domains) were modeled as indicators of a higher-order latent construct representing job stress. Model identification was achieved by fixing latent variances, and standardized solutions were reported. Standardized factor loadings, together with associated test statistics, *p*-values, and item-level coefficients of determination (R^2^), were used to assess the adequacy of the measurement model. The standardized factor loadings (λ) were interpreted as follows: λ ≥ 0.70 indicates a strong indicator; 0.50–0.69 is moderate; 0.30–0.49 is weak; λ < 0.30 is considered below the threshold for meaningful interpretation. For clarity, the factor structure was additionally presented in the form of a path diagram.

To examine measurement invariance across groups, configural, metric, and scalar multi-group CFA models were estimated for the higher-order job stress construct, with the seven composite scales serving as indicators. Invariance was evaluated by comparing nested models using chi-square difference tests (configural vs. metric; metric vs. scalar). To further assess potential item-level differential item functioning (DIF), score tests were conducted under the scalar model to examine the equality of intercepts. Items with significant results were flagged as exhibiting uniform DIF, indicating non-invariant intercepts across groups. All analyses were conducted using R software (version 4.4.3).

### 2.5. Ethical Considerations

The survey was performed in accordance with the Declaration of Helsinki. The Bioethics Committee of Klaipeda University approved the study protocol (Ref. No. 46Sv-VS-04, 8 November 2024).The consent form for participation was distributed to all participants and signed.

## 3. Results

Table 1 summarizes baseline characteristics for the overall study sample and report *p*-values for between-group comparisons by gender, years of service, and job position. Detailed differences between groups are described in the subsections below. Notably, within the “Change at work” domain, statistically significant differences were observed for all three comparisons (gender, years of service, and job position), aligning with the study aim to examine subgroup variation.

### 3.1. Gender

Gender-based comparisons across a set of work environment and job stress-related items were conducted. The total sample comprised 381 participants, of whom 206 were women (54.1%) and 175 were men (45.9%). In most cases, responses were highly similar between genders and effect sizes were negligible.

Significant differences emerged in the domain of “Change at work” and “Work control”. Men reported greater choice in how to perform work, and more say in work speed. They were also more likely to indicate experiencing unrealistic time pressures and to report opportunities to question managers about change, as well as greater flexibility of working time. The magnitude of these differences was consistent across variables, with effect sizes ranging between 0.13 and 0.16, corresponding to small effects; these findings should be interpreted cautiously.

### 3.2. Years of Service

When comparing employees with shorter (0–20 years, *n* = 205) versus longer (21+ years, *n* = 176) tenure, most work environment ratings were very similar between groups. Statistically significant differences appeared only for a subset of items, generally reflecting more positive evaluations among employees with longer service.

Specifically, respondents with over 21 years of service reported fewer unachievable deadlines, less unrealistic time pressure, and lower intensity of fast-paced work. They also indicated clearer understanding of how their work fits into the overall aims of the organization, greater opportunities to question managers about change, and more favorable perceptions of “Change at work” domain. In contrast, employees with shorter tenure more often reported higher demands.

For the remaining indicators—including managerial support, peer support, relationships, and role clarity—no significant differences emerged. Overall, observed effects were consistent in magnitude, with all statistically significant results corresponding to small effect sizes (r ≈ 0.10–0.15). Overall, effect sizes were small, indicating modest practical significance despite statistical significance in some comparisons.

### 3.3. Job Position

Comparisons between junior (*n* = 228) and senior (*n* = 153) employees revealed relatively few statistically significant differences. In most domains, responses were very similar across job positions, and effect sizes were small.

Significant differences appeared primarily in relation to control, organizational change, and managerial support. Junior employees reported greater ability to decide when to take a break, more favorable perceptions of departmental goals and objectives, more positive views on being consulted about organizational change, and greater flexibility of working time. They also expressed more positive perceptions of change at work and were more likely to feel encouraged by their line manager.

For other aspects—including demands, peer support, role clarity, relationships, and exposure to workplace stressors—differences between junior and senior staff were not significant, and associated effect sizes were negligible.

Overall, observed effects were consistent in magnitude, with all statistically significant findings corresponding to small effect sizes (r ≈ 0.11–0.15).

### 3.4. Analysis of Variance and Confirmatory Models

As a next step, univariate ANOVAs were fitted. Significant main effects of years of service emerged for Demands (*p* = 0.047), while job position was associated with Change at work (*p* = 0.031) and Work control (*p* = 0.037). Gender also influenced Work control (*p* = 0.010).

Several interactions were detected, including gender × years of service effects for Change at work (*p* = 0.015), Work control (*p* = 0.025), and Role clarity (*p* = 0.020). The gender × job position interaction reached significance for Peer support (*p* = 0.030). Additionally, the three-way interaction of gender × job position × years of service was significant for Work control (*p* = 0.005) and Role clarity (*p* = 0.006) (Table 2).

The global fit of CFA (Table 3) indices showed that the hypothesized second-order factor structure did not achieve conventionally accepted thresholds for good fit, resulting in methodological caution. The χ^2^ test was significant, suggesting deviations between the model and data. Incremental fit indices, specifically the CFI (0.768) and TLI (0.751), were substantially below the conventional guidelines of approximately 0.95. While the SRMR was acceptable at 0.079 (below the 0.08 threshold), the RMSEA of 0.085 (90% CI: 0.082–0.089) slightly exceeded the typical upper bound of 0.08. Overall, the indices point to a moderate but statistically inadequate fit of the proposed theoretical model to the observed data, an important limitation that challenges the full adequacy of the conceptual framework.

All first-order factor loadings were statistically significant (*p* < 0.001), supporting the convergent validity of the constructs. Within factors, most items demonstrated moderate to strong standardized loadings (0.55–0.83), with especially high values for Peer support (e.g., Item 24 = 0.880; Item 7 = 0.816) and Change at work (Item 26 = 0.828). In contrast, some items showed weaker loadings, such as Item 20 (Demands: 0.316) and Item 4 (Role clarity: 0.357), indicating limited explanatory power (R^2^ < 0.15). Despite these weaker indicators, factor-level reliability was acceptable and consistent with prior applications of the HSE-MSIT.

At the higher-order level, the Job stress factor was well represented by the seven domains. Standardized loadings were strong and statistically significant (*p* < 0.001), ranging from 0.659 (Role clarity) to 0.950 (Managerial support). The highest contributions came from Managerial support (0.950), Change at work (0.873), and Peer support (0.838), suggesting these aspects were most central to the global construct of job stress. Weaker but still meaningful contributions were observed for Role clarity (0.659) and Demands (0.675) (Table 4). This pattern underscores the predominance of organizational resources (leadership and change processes) within the higher-order stress construct.

Tests of measurement invariance showed that for gender and job position, the overall factor structure was comparable across groups (Table 5, Supplementary Materials Tables S1–S3). However, significant differences emerged at the level of item intercepts (uniform DIF), meaning that average scores should be compared with extreme caution. Notably, non-invariance was stronger for years of service, appearing already at earlier stages of testing, which indicates lower comparability of mean domain scores between shorter- and longer-tenure employees. These specific DIF findings challenge the validity of the univariate ANOVA results reported above.

## 4. Discussion

### 4.1. Main Findings

Across the full set of analyses, group differences in job stress and related work environment factors were present but consistently small in magnitude, with observed effect sizes typically ranging between r ≈ 0.10 and r ≈ 0.16. Employees with longer tenure generally described more favorable working conditions, including a clearer understanding of organizational goals and processes, and fewer experiences of unrealistic demands. Junior staff often evaluated managerial support and organizational change more positively than senior staff, though these differences were limited in scope.

The years of service and job position played a more prominent role than gender in shaping perceptions of the work environment, although several interaction effects indicated that group differences depend on combinations of demographic and occupational characteristics rather than single factors alone.

The CFA conceptually supported the representation of job stress as a higher-order construct with seven interrelated domains. However, it is important to note that the statistical fit of this second-order model was only moderate (CFI = 0.768; TLI = 0.751) and failed to meet conventional thresholds. Within this structure, Managerial support (loading 0.950), Change at work (0.873), and Peer support (0.838) emerged as the most central components of the global job stress construct, while the Demands (0.675) and Role clarity (0.659) domains contributed less strongly. These findings regarding centrality must therefore be interpreted with caution given the model’s overall statistical inadequacy. Model fit indices suggested that the global structure only moderately represented the data, implying that individual or cultural differences may affect how stress is perceived across groups. Tests of measurement invariance showed that, although the general structure of the model holds across gender and job position, some item intercepts vary across groups, and these differences are more pronounced for years of service. Accordingly, comparisons between groups should be interpreted as indicative trends rather than as definitive differences.

### 4.2. Analysis of Results

The study provides a clearer understanding of how Lithuanian police officers perceive and cope with work-related stress across different demographic and professional groups. The results showed some noticeable differences based on years of service and job position, while gender had less influence. This pattern corresponds with previous findings from other European countries, suggesting that organizational context and leadership climate are stronger predictors of stress than gender alone [16,29].

Gender-related differences in stress perception were relatively minor, which is consistent with findings from other international studies indicating that men and women in policing generally experience comparable overall stress levels. Male officers reported having slightly more autonomy in organizing their tasks, a greater ability to adjust their work pace, and more opportunities to provide feedback regarding organizational changes.

However, it must be acknowledged that the magnitude of these differences was consistently small (effect sizes r ≈ 0.13–0.16), suggesting that while statistically significant, these differences hold limited practical significance for large-scale intervention planning. These findings suggest that men may perceive higher job control, which, while potentially protective against certain stressors, can also be linked to increased workload and time pressure. In contrast, female officers often face additional challenges related to work-family balance and emotional demands, which, although not pronounced in the current sample, have been identified as important sources of strain in prior studies [29,35]. Consistent with other findings in this study, the statistically significant differences observed between tenure groups were also characterized by small effect sizes (r ≈ 0.10–0.15), warranting cautious interpretation of their practical impact. The relatively small gender gap may reflect the progressive gender integration policies within the Lithuanian police, where women have increasingly taken on operational and leadership roles.

Officers in junior roles tended to rely more on peer networks and were especially receptive to managerial feedback, reflecting their early stage of professional adjustment. In contrast, senior officers more often reported stress stemming from administrative workload, leadership obligations, and greater accountability—findings consistent with other European studies [16,28].

When examining differences by years of service, a slightly different pattern emerged. Officers with longer professional experience tended to adapt more easily to job demands and demonstrated clearer understanding of their roles and organizational expectations. This finding aligns with previous research indicating that accumulated experience enhances coping capacity and emotional regulation. Conversely, less experienced officers reported greater uncertainty, role confusion, and limited autonomy in decision-making—results that mirror international findings. Importantly, junior officers also reported receiving stronger managerial guidance and feedback, suggesting that supervisors may offer more direct support to newly integrated staff, as part of early-career adaptation [21,22,23].

In the Lithuanian context, these findings may also reflect the effects of organizational reforms implemented over the past decade, which have introduced new management standards, performance evaluation systems, and communication protocols within the national police. Such structural changes can temporarily increase uncertainty but also promote transparency and accountability once internal processes stabilize.

Peer and organizational support emerged as particularly strong protective factors against occupational stress. This finding is consistent with prior research demonstrating that structured peer-support programs can reduce stress while strengthening resilience and trust among police officers. Canadian officers emphasized that peer-support initiatives fostered a sense of belonging and open communication, helping them process traumatic experiences more effectively [36]. Similarly, the results of the present study indicate that Lithuanian police officers who receive consistent encouragement and assistance from both supervisors and colleagues report lower stress levels and greater overall morale, underscoring the importance of supportive professional environments within law enforcement institutions.

A comprehensive review, which analyzed 36 studies published between 1983 and 2022, emphasized the critical importance of organizational support in mitigating occupational stress among police officers. The authors concluded that a supportive organizational climate is closely linked to improved mental health and reduced job strain. Moreover, active involvement of officers in decision-making processes and transparent communication during periods of organizational change were identified as key factors that enhance coping capacity and foster a sense of control. These findings are consistent with results from other studies, which have similarly demonstrated that strong organizational support, fair leadership, and open communication significantly reduce stress and emotional exhaustion among police officers [7,37,38]. Importantly, these findings reinforce the view that job stress among police personnel is rooted more in systemic and organizational dynamics than in individual vulnerabilities. In smaller systems such as Lithuania’s, where resources and staff numbers are limited, transparent communication, fair supervision, and peer collaboration may be particularly critical for sustaining resilience.

In recent years, more attention has been given to how stress among police officers can be prevented rather than only managed once it occurs. Research shows that emotional and social support within an organization plays a crucial role in protecting officers’ mental health and improving their ability to cope with work-related challenges. Peer-support programs are particularly effective, as they help create a sense of belonging, encourage open communication, and strengthen trust between colleagues. In addition, mindfulness-based approaches—such as short reflective breaks, gratitude or compassion exercises, and other simple awareness techniques—have proven useful in restoring emotional balance and reducing tension in demanding jobs [36,37,39,40].

Overall, the results of this study are in line with international findings, suggesting that police stress cannot be reduced by a single measure. Instead, a combination of organizational fairness, strong peer connections, and supportive leadership is most effective in helping officers handle everyday pressures and maintain long-term psychological well-being.

### 4.3. Stress Management in Police Officers

Based on the findings of this study, several strategies are proposed to reduce occupational stress and promote psychological well-being among police officers. These recommendations address both organizational and individual levels of intervention, aiming to foster a healthier and more supportive work environment.

To enhance officers’ psychological resilience, institutions are encouraged to implement structured peer-support programs. Such programs could involve experienced officers trained to provide emotional and psychological assistance to colleagues, helping to create a culture of trust, mutual care, and early stress recognition.

Reducing occupational stress also requires improving emotional literacy within police organizations. Regular workshops, seminars, and training sessions focused on emotion recognition, regulation, and communication can help officers manage stress more effectively and strengthen interpersonal relationships at work.

Furthermore, as Managerial support was the strongest factor in the CFA model, targeted interventions directed to this area are particularly warranted. In addition to general well-being or resilience-building programs, police agencies should implement specialized training for managers to enhance their ability to recognize, prevent, and manage occupational stress among subordinates. Such training should include modules on supportive leadership and communication, early recognition of stress symptoms, and constructive feedback practices that help create a climate of psychological safety. In addition, managers should be equipped with coaching and mentoring tools that promote engagement, trust, and transparent communication during organizational change.

Empirical research in high-stress professional environments confirms that leadership interventions focused on strengthening supervisor support significantly reduce burnout, absenteeism, and turnover intentions. Therefore, providing structured training to line managers may be one of the most cost-effective strategies to enhance organizational well-being and long-term resilience in the context of policing.

To mitigate interpersonal conflicts and workplace tension, institutions should promote informal team-building activities and social events that strengthen cooperation and collegiality. It is equally important to ensure fair, transparent, and clearly defined work distribution, which contributes to a sense of justice and organizational trust.

For stress prevention, integrating natural elements into the workplace is highly recommended. Short, regular breaks—such as five-minute pauses each hour—allow officers to observe or interact with restorative natural features, such as indoor plants, outdoor scenery, or nature imagery. Additionally, officers could be encouraged to take brief walks in natural surroundings and to practice mindfulness techniques. Simple practices, such as pausing outdoors, consciously relaxing, and focusing on sounds like birdsong or rustling leaves, can help restore attention, reduce tension, and promote emotional balance.

### 4.4. Strengths and Limitations

This study broadens the understanding of occupational stress among police officers—one of the most psychologically demanding professions. It provides new insights into the patterns of work-related stress in this field and highlights areas that could inform effective stress prevention and management strategies. By identifying specific stressors, the study contributes to improving occupational health, safety, and overall well-being within the police force. Another important strength lies in the use of a validated measurement instrument (the HSE Management Standards Indicator Tool) and the focus on a relatively under-researched population in the Baltic region, which adds value to the international literature on occupational stress in law enforcement.

Like all research, this study has several limitations. The relatively small sample size may not fully capture the diversity of stress experiences among Lithuanian police officers. Expanding the study geographically—to include participants from other Baltic or Nordic countries—could provide a more comprehensive view of how work-related stress manifests across different cultural and organizational contexts. Differences in national working conditions, support systems, and approaches to stress prevention may also influence how officers perceive and respond to stress, making cross-country comparisons valuable for future research.

A further critical constrain on the practical interpretation of our findings is that all observed statistically significant group differences (derived from ANOVA and individual item comparisons) were associated with small effect sizes (ranging from r ≈ 0.10 to r ≈ 0.16). While these results confirm the existence of group variations, the small magnitude implies that the reported differences have limited explanatory power and may not translate into substantial differences in actual stress experience or substantial practical implications for broad intervention development. Therefore, our interpretations regarding group differences must focus primarily on the patterns of influence (e.g., years of service having a stronger role than gender) rather than the size of the effect.

Another limitation concerns the absence of a detailed analysis of job design. Working environments differ considerably between officers with predominantly indoor duties and those working in the field, and such variations may influence stress exposure and coping mechanisms. Future research should therefore explore how the nature of police work and its organizational design affect stress experiences.

Although overall stress patterns appear broadly similar across subgroups, direct score comparisons, particularly those derived from the ANOVA analysis, must be interpreted with extreme caution. This is due to the detection of selective item-level non-invariance (DIF) across gender, job position, and, most prominently, years of service.

The presence of DIF indicates that the underlying construct of occupational stress is not measured identically across these groups. Specifically, the observed differences at the item intercept level (Table 5) mean that the reported mean-level differences (e.g., in Demands, Work control, or Change at work) found via ANOVA may be biased or partly reflect differences in how respondents interpret or understand specific survey items, rather than genuine differences in true stress levels.

For instance, items showing DIF in the *Years of service* comparison (such as Item 23: *I can rely on my line manager...* or Item 24: *I get help and support I need from colleagues*) suggest that longer-tenure officers may respond more positively to these questions than less experienced officers, not necessarily because they receive objectively better support, but potentially because they interpret the concept of ‘support’ differently or have adjusted their expectations.

Therefore, the statistically significant ANOVA results, particularly the main effects (Demands by years of service; Change at work by job position; Work control by gender and job position), cannot be taken as definitive evidence of substantive group differences, but rather as indicators of areas where the instrument’s validity and cross-group comparability are compromised. Future studies should address this by employing DIF-adjusted scores (e.g., using latent mean comparison models).

Finally, the cross-sectional nature of this research restricts causal inferences. The findings are based on data collected at one point in time, preventing conclusions about changes or trends over the course of an officer’s career. Longitudinal or mixed-method studies could offer a deeper understanding of how occupational stress develops, persists, and can be effectively mitigated over time.

A significant methodological limitation lies in the fit of the second-order Confirmatory Factor Analysis (CFA) model. The model’s incremental fit indices (CFI = 0.768, TLI = 0.751) were substantially below the conventionally accepted thresholds for a good fit (e.g., 0.95). This inadequate fit indicates that the proposed hierarchical theoretical structure—where the seven first-order domains (Demands, Managerial support, Peer support, Relationships, Change at work, Work control, Role clarity) fully converge into a single, higher-order ‘Job Stress’ construct—is only moderately supported by the data in this specific population. The poor fit challenges the full adequacy of the adopted theoretical framework as applied here, suggesting that the underlying structure of occupational stress among Lithuanian police officers may be more complex or non-hierarchical.

Furthermore, the failure to meet robust fit criteria means that the high magnitude of the second-order loadings, such as Managerial support (0.950), should be interpreted with statistical restraint. To fully address this methodological concern, future research should test and report on alternative model specifications. Potential alternatives include examining a first-order oblique model without a higher-order factor, or testing models that posit multiple correlated latent higher-order constructs, which may provide a statistically better fit and a more accurate representation of stress patterns in this context.

A notable limitation of this study is that data were collected exclusively through written questionnaires. This approach may introduce self-report bias, as respondents could provide socially desirable answers. It also limits the depth of insights because there was no opportunity to clarify questions or explore responses further. Non-response bias is possible if officers who declined participation differ systematically from those who participated. Additionally, the formal setting of data collection may have influenced the openness of responses, and the cross-sectional design restricts the ability to assess changes over time or establish causal relationships.

### 4.5. Future Research

Future research should build on these findings by exploring not only the sources of occupational stress but also its consequences. Studies could incorporate measures of sleep quality, nutrition, and mental health outcomes such as anxiety, depression, or burnout. Expanding research geographically to include police officers from other Baltic and Nordic countries would also provide opportunities for cross-cultural comparison and highlight regional differences in stress perception and management.

Additionally, examining job design and the specific nature of policing tasks—such as differences between field and administrative roles—could clarify how work environments influence stress levels. Mixed-method approaches, combining quantitative assessment with qualitative interviews, would allow for a more nuanced understanding of how officers experience and cope with stress in daily practice. Such research could inform targeted interventions, guide policy development, and strengthen long-term well-being among police personnel.

## 5. Conclusions

This study identified consistent yet small differences in occupational stress patterns among Lithuanian police officers, with years of service and job position exerting a greater influence than gender. Officers with longer tenure reported fewer unrealistic demands and a clearer understanding of organizational goals, whereas junior staff relied more heavily on managerial support and peer networks. Gender-based differences were minimal, suggesting broadly similar stress experiences between men and women. However, the moderate fit of the confirmatory factor model indicates that these relationships should be interpreted with caution, as cultural and organizational factors may influence how stress is perceived and reported. Importantly, confirmatory factor analyses indicated that managerial support, peer support, and organizational change represent the most central protective domains within the broader construct of occupational stress.

The findings emphasize that organizational and interpersonal dynamics outweigh individual characteristics in shaping police officers’ experiences of stress. Supportive leadership, transparent communication, and opportunities for participation in decision-making emerged as critical determinants of reduced stress levels. Peer networks also played a highly protective role, underscoring the importance of collegial trust and social connectedness within police institutions. These findings align with broader European research highlighting that, in smaller and reform-oriented systems such as Lithuania’s, the quality of leadership and communication plays a decisive role in maintaining officers’ psychological well-being.

Practical recommendations include the implementation of structured peer-support systems led by trained officers, the expansion of emotional literacy training, and the integration of mindfulness and restorative practices into daily routines. Institutions should also ensure fair workload distribution, strengthen supervisory support, and foster open communication during periods of organizational change. Regular workshops, informal team-building, and short restorative breaks involving natural elements may further enhance resilience and emotional well-being.

Overall, the study suggests that police stress cannot be effectively addressed through a single intervention. Instead, a comprehensive approach combining organizational fairness, strong peer relationships, and evidence-based psychological strategies is needed. Future research should aim to validate these findings longitudinally and explore intervention outcomes in comparable occupational settings, such as healthcare or emergency services. By prioritizing these measures, law enforcement agencies can reduce occupational strain, safeguard officers’ mental health, and enhance long-term institutional performance.

## Figures and Tables

**Table 1:** Comparison of domains and items by gender, years of service and job position.

Variable (Item)	Overall (N = 381)			*p*-Value		
	Mean (SD)	Median (Q1-Q3)	Range	Female vs. Male	0–20 Years of Service vs. 21+ Years of Service	Junior vs. Senior
**Demands**	26.76 (4.6)	27 (24–30)	8–38	0.117	**0.031**	0.795
Different groups at work demand things from me that are hard to combine (3)	3.47 (0.92)	3 (3–4)	1–5	0.256	0.111	0.370
I have unachievable deadlines (6)	3.66 (0.91)	5 (4–5)	1–5	0.806	**0.017**	0.477
I have to work very intensively (9)	2.44 (0.76)	3 (2–3)	1–5	0.053	0.683	0.824
I have to neglect some tasks because I have too much to do (12)	3.50 (0.86)	3 (3–4)	1–5	0.253	0.793	0.499
I am unable to take sufficient breaks (16)	3.48 (0.94)	3 (3–4)	1–5	0.462	0.235	0.417
I am pressured to work long hours (18)	4.16 (0.98)	4 (4–5)	1–5	0.375	0.977	0.909
I have to work very fast (20)	2.54 (0.89)	3 (2–3)	1–5	0.265	**0.011**	0.988
I have unrealistic time pressures (22)	3.51 (0.98)	3 (3–4)	1–5	**0.004**	**0.020**	0.133
**Managerial support**	17.21 (4.23)	17 (15–21)	5–25	0.306	0.852	0.068
I am given supportive feedback on the work I do (8)	3.38 (0.94)	3 (3–4)	1–5	0.253	0.793	0.499
I can rely on my line manager to help me out with a work problem (23)	3.87 (1.00)	4 (3–5)	1–5	0.912	**0.011**	0.699
I can talk to my line manager about something that has upset or annoyed me about work (29)	3.52 (1.26)	4 (3–5)	1–5	0.108	0.291	0.067
I am supported through emotionally demanding work (33)	3.19 (1.01)	3 (3–4)	1–5	0.799	0.877	0.218
My line manager encourages me at work (35)	3.24 (0.99)	3 (3–4)	1–5	0.084	0.733	**0.003**
**Peer support**	14.71 (3.1)	15 (12–17)	4–20	0.902	0.928	0.771
If work gets difficult, my colleagues will help me (7)	3.72 (0.98)	4 (3–4)	1–5	0.743	0.901	0.972
I get help and support I need from colleagues (24)	3.78 (0.89)	4 (3–4)	1–5	0.652	0.151	0.540
I receive the respect at work I deserve from my colleagues (27)	3.59 (0.84)	4 (3–4)	1–5	0.868	0.158	0.662
My colleagues are willing to listen to my work-related problems (31)	3.62 (0.93)	4 (3–4)	1–5	0.895	0.888	0.183
**Relationships**	15.23 (2.71)	16 (14–17)	4–20	0.615	0.910	0.804
I am subject to personal harassment in the form of unkind words or behavior (5)	4.24 (0.99)	5 (4–5)	1–5	0.618	0.974	0.925
There is friction or anger between colleagues (14)	3.15 (0.82)	3 (3–4)	1–5	0.586	0.977	0.982
I am subject to bullying at work (21)	4.41 (0.93)	5 (4–5)	1–5	0.572	0.553	0.837
Relationships at work are strained (34)	3.43 (0.93)	3 (3–4)	1–5	0.273	0.789	0.848
**Change at work**	9.87 (2.61)	10 (8–12)	3–15	**0.009**	**0.005**	**0.019**
I have sufficient opportunities to question managers about change at work (26)	3.69 (1.07)	4 (3–4)	1–5	**0.011**	**0.025**	0.323
Staff are always consulted about change at work (28)	3.12 (1.12)	3 (2–4)	1–5	0.184	0.057	**0.036**
When changes are made at work, I am clear how they will work out in practice (32)	3.06 (0.98)	3 (3–4)	1–5	0.061	**0.039**	0.082
**Work control**	20.97 (4.26)	21 (18–24)	6–30	**0.002**	0.296	0.064
I can decide when to take a break (2)	4.00 (0.79)	4 (4–5)	1–5	0.519	0.217	**0.014**
I have a say in my own work speed (10)	3.45 (1.1)	3 (3–4)	1–5	**0.003**	0.816	0.099
I have a choice in deciding how I do my work (15)	3.56 (0.88)	4 (3–4)	1–5	**0.005**	0.150	0.808
I have a choice in deciding what I do at work (19)	3.56 (0.88)	4 (3–4)	1–5	**0.005**	0.150	0.808
I have some say over the way I work (25)	3.05 (1.1)	3 (2–4)	1–5	0.115	0.220	0.114
My working time can be flexible (30)	3.10 (1.16)	3 (2–4)	1–5	**0.010**	**0.019**	**0.025**
**Role clarity**	21.52 (2.34)	22 (20–23)	13–25	0.692	0.057	0.217
I am clear what is expected of me at work (1)	4.32 (0.65)	4 (4–5)	1–5	0.631	0.383	0.538
I know how to go about getting my job done (4)	4.27 (0.51)	4 (4–5)	2–5	0.110	0.148	0.749
I am clear what my duties and responsibilities are (11)	4.49 (0.72)	5 (4–5)	1–5	**0.003**	0.816	0.099
I am clear about the goals and objectives for my department (13)	4.29 (0.71)	5 (4–5)	1–5	0.310	0.249	**0.033**
I understand how my work fits into the overall aim of the organization (17)	4.15 (0.72)	4 (4–5)	2–5	0.518	**0.009**	0.303

**Table 2:** Statistically significant ANOVA results.

Dependent Variable	Effect	Statistic	*p*-Value
Demands	Years of service	3.977	0.047
Peer support	Gender × Job position	4.717	0.030
Peer support	Gender × Years of service	9.092	0.003
Change at work	Job position	4.666	0.031
Change at work	Gender × Years of service	5.961	0.015
Work control	Gender	6.796	0.010
Work control	Job position	4.388	0.037
Work control	Gender × Years of service	5.041	0.025
Work control	Gender × Job position × Years of service	7.920	0.005
Role clarity	Gender × Years of service	5.478	0.020
Role clarity	Gender × Job position × Years of service	7.558	0.006

**Table 3:** Confirmatory Factor Analysis—Standardized loadings (first-order) with Statistic (z), *p*, and R^2^.

Factor	Item	Loading	Statistic (z)	*p*-Value	R^2^
Change at work	Item 26	0.828	9.79	<0.001	0.686
Change at work	Item 28	0.760	9.73	<0.001	0.578
Change at work	Item 32	0.565	8.29	<0.001	0.319
Demands	Item 12	0.433	7.95	<0.001	0.188
Demands	Item 16	0.682	12.87	<0.001	0.465
Demands	Item 18	0.704	13.32	<0.001	0.496
Demands	Item 20	0.316	5.73	<0.001	0.100
Demands	Item 22	0.753	14.30	<0.001	0.567
Demands	Item 3	0.545	10.12	<0.001	0.297
Demands	Item 6	0.617	11.56	<0.001	0.381
Demands	Item 9	0.458	8.43	<0.001	0.210
Managerial support	Item 23	0.743	6.29	<0.001	0.552
Managerial support	Item 29	0.789	6.35	<0.001	0.622
Managerial support	Item 33	0.795	6.36	<0.001	0.632
Managerial support	Item 35	0.717	6.25	<0.001	0.515
Managerial support	Item 8	0.715	6.24	<0.001	0.511
Peer support	Item 24	0.880	14.66	<0.001	0.774
Peer support	Item 27	0.687	12.05	<0.001	0.472
Peer support	Item 31	0.802	13.75	<0.001	0.643
Peer support	Item 7	0.816	13.94	<0.001	0.666
Relationships	Item 14	0.650	9.36	<0.001	0.423
Relationships	Item 21	0.623	9.12	<0.001	0.388
Relationships	Item 34	0.646	9.32	<0.001	0.417
Relationships	Item 5	0.559	8.48	<0.001	0.313
Role clarity	Item 1	0.641	11.52	<0.001	0.411
Role clarity	Item 11	0.672	12.07	<0.001	0.451
Role clarity	Item 13	0.759	13.44	<0.001	0.576
Role clarity	Item 17	0.557	9.98	<0.001	0.311
Role clarity	Item 4	0.357	6.31	<0.001	0.127
Work control	Item 10	0.710	13.37	<0.001	0.505
Work control	Item 15	0.748	14.10	<0.001	0.560
Work control	Item 19	0.678	12.74	<0.001	0.460
Work control	Item 2	0.611	11.40	<0.001	0.373
Work control	Item 25	0.761	14.35	<0.001	0.579
Work control	Item 30	0.477	8.80	<0.001	0.227

**Table 4:** Confirmatory Factor Analysis—Second-order loadings.

Higher Order	First Order	Loading	Statistic (z)	*p*-Value
Job Stress	Change at work	0.873	8.45	<0.001
Job Stress	Demands	0.675	10.05	<0.001
Job Stress	Managerial support	0.950	5.91	<0.001
Job Stress	Peer support	0.838	11.03	<0.001
Job Stress	Relationships	0.806	8.50	<0.001
Job Stress	Role clarity	0.659	9.35	<0.001
Job Stress	Work control	0.718	10.53	<0.001

**Table 5:** Item-level differential item functioning—gender, job position and years of service (intercepts).

Effect	Item	Statistic	*p*-Value
Gender	Item 22	8.19	0.004
Gender	Item 3	7.94	0.004
Gender	Item 33	5.72	0.017
Gender	Item 13	3.98	0.046
Job position	Item 23	7.92	0.005
Job position	Item 15	6.64	0.009
Job position	Item 35	6.25	0.012
Job position	Item 30	4.31	0.038
Job position	Item 2	3.97	0.046
Years of service	Item 23	12.84	<0.001
Years of service	Item 19	10.32	0.001
Years of service	Item 24	7.91	0.005
Years of service	Item 18	5.33	0.021
Years of service	Item 27	5.23	0.022
Years of service	Item 30	4.40	0.036
Years of service	Item 20	3.94	0.047

## Data Availability

The data are not publicly available due to confidentiality and privacy considerations. In addition, restrictions apply to the availability of these data due to our policy statement of sharing data. Data are available only on reasonable request.

## Supplementary Materials

The following supporting information can be downloaded at: https://www.mdpi.com/article/10.3390/healthcare13233077/s1, Table S1: Item-level differential item functioning—Gender (intercepts); Table S2: Item-level differential item functioning—Job position (intercepts); Table S3: Item-level differential item functioning—Years of service (intercepts).
