# Supplementary material for: Patterns of Self-Reported Occupational Stress Experienced by Lithuanian Police Officers: A Cross-Sectional Study
Source: Healthcare (Basel). 2025 Nov 26;13(23):3077. doi: 10.3390/healthcare13233077 (PMC12691733; doi:10.3390/healthcare13233077)
Supplement: Supplementary file 1 [file healthcare-13-03077-s001.zip › healthcare-3945970-supplementary.pdf]

Supplementary Materials

Table S1. Item-level differential item functioning – Gender (intercepts)

Effect	Item	Statistic	p-value
Gender	Item 22	8.19	**0.004**
Gender	Item 3	7.94	**0.004**
Gender	Item 33	5.72	**0.017**
Gender	Item 13	3.98	**0.046**
Gender	Item 2	3.47	0.062
Gender	Item 4	2.82	0.093
Gender	Item 35	2.71	0.099
Gender	Item 5	2.64	0.104
Gender	Item 25	2.23	0.135
Gender	Item 15	2.19	0.139
Gender	Item 28	2.01	0.157
Gender	Item 26	1.84	0.174
Gender	Item 30	1.68	0.194
Gender	Item 16	1.28	0.258
Gender	Item 6	1.17	0.280
Gender	Item 9	1.14	0.285
Gender	Item 29	1.11	0.291
Gender	Item 10	1.05	0.304
Gender	Item 8	0.89	0.345
Gender	Item 34	0.80	0.370
Gender	Item 23	0.79	0.374
Gender	Item 20	0.52	0.469
Gender	Item 24	0.45	0.501
Gender	Item 1	0.40	0.525
Gender	Item 17	0.32	0.574
Gender	Item 7	0.25	0.615
Gender	Item 14	0.20	0.655
Gender	Item 11	0.17	0.676
Gender	Item 19	0.11	0.738
Gender	Item 12	0.07	0.792
Gender	Item 18	0.07	0.789
Gender	Item 27	0.04	0.849
Gender	Item 31	0.02	0.896
Gender	Item 21	0.00	0.990
Gender	Item 32	0.00	0.979

Table S2. Item-level differential item functioning – Job position (intercepts)

Effect	Item	Statistic	p-value
Job position	Item 23	7.92	**0.005**
Job position	Item 15	6.64	**0.009**
Job position	Item 35	6.25	**0.012**
Job position	Item 30	4.31	**0.038**
Job position	Item 2	3.97	**0.046**
Job position	Item 19	3.68	0.055
Job position	Item 22	3.29	0.070

Job position	Item 3	2.95	0.086
Job position	Item 27	2.08	0.149
Job position	Item 4	1.68	0.194
Job position	Item 26	1.64	0.201
Job position	Item 12	1.45	0.229
Job position	Item 31	1.42	0.234
Job position	Item 10	1.03	0.309
Job position	Item 7	0.78	0.377
Job position	Item 32	0.74	0.389
Job position	Item 29	0.72	0.395
Job position	Item 13	0.63	0.428
Job position	Item 11	0.59	0.443
Job position	Item 28	0.58	0.448
Job position	Item 14	0.44	0.507
Job position	Item 25	0.43	0.514
Job position	Item 1	0.39	0.531
Job position	Item 24	0.39	0.533
Job position	Item 5	0.35	0.551
Job position	Item 9	0.32	0.57
Job position	Item 17	0.14	0.710
Job position	Item 16	0.13	0.720
Job position	Item 8	0.13	0.716
Job position	Item 18	0.06	0.803
Job position	Item 33	0.03	0.868
Job position	Item 34	0.01	0.907
Job position	Item 20	0.00	0.953
Job position	Item 21	0.00	0.980
Job position	Item 6	0.00	0.953

Table S3. Item-level differential item functioning – Years of service (intercepts)

Effect	Item	Statistic	p-value
Years of service	Item 23	12.84	**<0.001**
Years of service	Item 19	10.32	**0.001**
Years of service	Item 24	7.91	**0.005**
Years of service	Item 18	5.33	**0.021**
Years of service	Item 27	5.23	**0.022**
Years of service	Item 30	4.40	**0.036**
Years of service	Item 20	3.94	**0.047**
Years of service	Item 29	3.50	0.061
Years of service	Item 17	3.15	0.076
Years of service	Item 6	2.98	0.084
Years of service	Item 12	2.24	0.134
Years of service	Item 11	1.92	0.166
Years of service	Item 22	1.78	0.182

Years of service	Item 10	1.30	0.254
Years of service	Item 15	1.07	0.301
Years of service	Item 2	0.89	0.347
Years of service	Item 8	0.78	0.377
Years of service	Item 31	0.72	0.395
Years of service	Item 25	0.60	0.440
Years of service	Item 9	0.57	0.449
Years of service	Item 7	0.50	0.482
Years of service	Item 4	0.28	0.599
Years of service	Item 13	0.24	0.622
Years of service	Item 33	0.21	0.645
Years of service	Item 16	0.20	0.657
Years of service	Item 3	0.16	0.686
Years of service	Item 14	0.14	0.706
Years of service	Item 5	0.14	0.704
Years of service	Item 28	0.13	0.716
Years of service	Item 32	0.07	0.788
Years of service	Item 1	0.03	0.859
Years of service	Item 26	0.03	0.867
Years of service	Item 35	0.03	0.868
Years of service	Item 34	0.02	0.893
Years of service	Item 21	0.01	0.941
